# Inhibition of Long-Term Variability in Decoding Forelimb Trajectory Using Evolutionary Neural Networks With Error-Correction Learning

**DOI:** 10.3389/fncom.2020.00022

**Published:** 2020-03-31

**Authors:** Shih-Hung Yang, Han-Lin Wang, Yu-Chun Lo, Hsin-Yi Lai, Kuan-Yu Chen, Yu-Hao Lan, Ching-Chia Kao, Chin Chou, Sheng-Huang Lin, Jyun-We Huang, Ching-Fu Wang, Chao-Hung Kuo, You-Yin Chen

**Affiliations:** ^1^Department of Mechanical Engineering, National Cheng Kung University, Tainan, Taiwan; ^2^Department of Biomedical Engineering, National Yang Ming University, Taipei, Taiwan; ^3^The Ph.D. Program for Neural Regenerative Medicine, College of Medical Science and Technology, Taipei Medical University, Taipei, Taiwan; ^4^Key Laboratory of Medical Neurobiology of Zhejiang Province, Department of Neurology of the Second Affiliated Hospital, Interdisciplinary Institute of Neuroscience and Technology, Zhejiang University School of Medicine, Hangzhou, China; ^5^Key Laboratory of Biomedical Engineering of Ministry of Education, College of Biomedical Engineering and Instrument Science, Qiushi Academy for Advanced Studies, Zhejiang University, Hangzhou, China; ^6^Research Center for Information Technology Innovation, Academia Sinica, Taipei, Taiwan; ^7^Department of Regulatory & Quality Sciences, University of Southern California, Los Angeles, CA, United States; ^8^Buddhist Tzu Chi Medical Foundation, Department of Neurology, Hualien Tzu Chi Hospital, Hualien, Taiwan; ^9^Department of Neurology, School of Medicine, Tzu Chi University, Hualien, Taiwan; ^10^Department of Neurosurgery, Neurological Institute, Taipei Veterans General Hospital, Taipei, Taiwan; ^11^Department of Neurological Surgery, University of Washington, Seattle, WA, United States

**Keywords:** brain machine interfaces, neural decoding, error feedback, evolutionary algorithm, recurrent neural network

## Abstract

**Objective:** In brain machine interfaces (BMIs), the functional mapping between neural activities and kinematic parameters varied over time owing to changes in neural recording conditions. The variability in neural recording conditions might result in unstable long-term decoding performance. Relevant studies trained decoders with several days of training data to make them inherently robust to changes in neural recording conditions. However, these decoders might not be robust to changes in neural recording conditions when only a few days of training data are available. In time-series prediction and feedback control system, an error feedback was commonly adopted to reduce the effects of model uncertainty. This motivated us to introduce an error feedback to a neural decoder for dealing with the variability in neural recording conditions.

**Approach:** We proposed an evolutionary constructive and pruning neural network with error feedback (ECPNN-EF) as a neural decoder. The ECPNN-EF with partially connected topology decoded the instantaneous firing rates of each sorted unit into forelimb movement of a rat. Furthermore, an error feedback was adopted as an additional input to provide kinematic information and thus compensate for changes in functional mapping. The proposed neural decoder was trained on data collected from a water reward-related lever-pressing task for a rat. The first 2 days of data were used to train the decoder, and the subsequent 10 days of data were used to test the decoder.

**Main Results:** The ECPNN-EF under different settings was evaluated to better understand the impact of the error feedback and partially connected topology. The experimental results demonstrated that the ECPNN-EF achieved significantly higher daily decoding performance with smaller daily variability when using the error feedback and partially connected topology.

**Significance:** These results suggested that the ECPNN-EF with partially connected topology could cope with both within- and across-day changes in neural recording conditions. The error feedback in the ECPNN-EF compensated for decreases in decoding performance when neural recording conditions changed. This mechanism made the ECPNN-EF robust against changes in functional mappings and thus improved the long-term decoding stability when only a few days of training data were available.

## Introduction

Brain machine interface (BMI) technology converts the brain's neural activity into kinematic parameters of limb movements. This allows controlling a computer cursor or prosthetic devices (Kao et al., 2014; Slutzky, 2018), which can greatly improve the quality of life. Intracortical BMIs have used microelectrodes implanted in the cortex to decode neural signals. These signals have then been converted into motor commands to control an anthropomorphic prosthetic limb, thereby restoring natural function (Collinger et al., 2013; Roelfsema et al., 2018).

The decoder was the most crucial component of a BMI; it modeled the functional mapping between neural activities and kinematic parameters (e.g., movement velocity or position), and assumed that this functional mapping was time-invariant (i.e., a stationary statistical assumption) (Kim et al., 2006). However, under real neural recording conditions, there existed a high degree of within- and across-day variability (Simeral et al., 2011; Perge et al., 2013, 2014; Wodlinger et al., 2014; Downey et al., 2018) that prevented satisfaction of the stationary statistical assumption. This variability consisted of the relative position of the recording electrodes—and surrounding neurons, electrode properties, tissue reaction to electrodes, and neural plasticity—and might affect the functional mapping between neural activities and kinematic parameters (Jackson et al., 2006; Barrese et al., 2013; Fernández et al., 2014; Salatino et al., 2017; Michelson et al., 2018; Hong and Lieber, 2019). The variability in neural recording conditions resulted in unstable long-term decoding performance and led to frequent decoder retraining (Jarosiewicz et al., 2013, 2015).

Conventional decoder retraining required the subject to periodically perform a well-defined task to collect new training data for preventing model staleness (Jarosiewicz et al., 2015). This manner may lead to additional training time before the BMI can be used. Traditional linear neural decoders did not need frequent retraining but possessed limited computational complexity to deal with neural recording condition changes owning to linear properties (Collinger et al., 2013). It is known that the newly encountered neural recording conditions in chronic BMI systems have some commonality with past neural recording conditions (Chestek et al., 2011; Perge et al., 2013; Bishop et al., 2014; Nuyujukian et al., 2014; Orsborn et al., 2014). Therefore, computationally powerful non-linear decoders were proposed to learn a diverse set of neural-to-kinematic mappings corresponding to various neural recording conditions collected over many days before BMI use (Sussillo et al., 2016). This approach avoided BMI interruption by keeping model parameters fixed during BMI use and made BMI inherently robust to changes in neural recording conditions by exploiting the similarities between newly encountered and past neural recording conditions. Therefore, the BMIs were trained with several days of data in order to learn various neural recording conditions and achieve stable long-term decoding. However, they heavily relied on the huge training data where a large training set may not be available for both non-human primates and rodent models.

The limited training data have become an issue for BMI application in long-term performance. A chronic inflammatory reaction results in neural signal loss and decrease in quality over time (Chen et al., 2009). Also, the number of implanted electrodes is limited by the size of the neural nuclei in the rodent brain. Therefore, limited neurons and limited recording times lead to limited training data.

Rodent models with small numbers of implanted electrodes have been widely used to investigate state-of-the-art neural prostheses. Previous studies have demonstrated the decoding performances of various methods at the motor cortex (Zhou et al., 2010; Yang et al., 2016), somatosensory cortex (Pais-Vieira et al., 2013), and hippocampus (Tampuu et al., 2019) in rodent models. The results indicated that good decoding methods should be considerably more robust to small sample sizes caused by limited neurons or limited recording times. In general, a limited amount of training data made traditional decoding methods inaccurate, because they usually required a large number of neurons to achieve desirable levels of performance. Furthermore, small amounts of data have made modern decoding methods unreliable, because their increasing model complexities required a large amount of training data (Glaser et al., 2017). Whether the BMIs could deal with the scenario in which only a few days of training data were available is unknown. This motivated us to develop a neural decoder that could learn from limited training data based on rodent models.

In time-series prediction applications, neural networks (NNs) usually employed prediction error as an additional input of the networks. This has been proven to yield superior performance compared with that without error feedback (Connor et al., 1994; Mahmud and Meesad, 2016; Waheeb et al., 2016). The error feedback determined the difference between the network output and the target value. This information could provide the network with information concerning previous prediction performance and might thereby guide the network to accurate prediction. In a feedback control system, the output signal was fed back to form an error signal, which was the difference between the target and actual output, in order to drive the system. Using feedback could reduce the effects of model uncertainty (Løvaas et al., 2008). Furthermore, feedback control could cope with trial-to-trial variability caused by complex dynamics or noise in motor behavior (Todorov and Jordan, 2002). Based on the contemporary physiological studies in the human cortex (Miyamoto et al., 1988), a feedback motor command has been used as an error signal for training an NN (Kawato, 1990). One study hypothesized that the user intended to directly move toward the target when using BMI. This study fitted the neural decoder by estimating user's intended velocity which was determined from target position, cursor position, and decoded velocity (Gilja et al., 2012). A recent study took into account how the user modified the neural modulation to deal with the movement errors caused by neural variability in the feedback loop (Willett et al., 2019). Their framework simulated online/closed-loop dynamics of an intracortical BMI and calibrated its decoder by an encoded control signal, which was the difference between target position and cursor position. The encoded control signal using target position was first transformed into neural features which were then mapped to a decoded control vector for updating decoder output, i.e., cursor velocity. This motivated us to introduce an error feedback into a neural decoder for dealing with the variability in neural recording conditions because the error feedback might compensate for the changes in neural recording conditions. Then, the neural decoder did not need retraining and was expected to be robust to various neural recording conditions when only using a few days of training data.

Several characteristics make NNs computationally powerful decoders in BMIs. First, an NN with a sufficient number of hidden neurons can approximate any continuous function (Hornik et al., 1989). This makes an NN well-suited to learn the functional mapping between neural activity and kinematic parameters. Second, several types of NNs can successfully control motor movement in BMIs. These include recurrent NN (RNN) (Haykin, 1994; Shah et al., 2019), echo-state network (ESN) (Jaeger and Haas, 2004), and time-delay NN (TDNN) (Waibel et al., 1989). RNNs have feedback connections that are capable of processing neural signal sequences. Their feedback loop is applicable to system dynamics modeling and time-dependent functional mapping between neural activity and kinematic parameters (Haykin, 1994). ESN was developed as an RNN that only trains connections between the hidden neurons and the output neurons for a simple learning process (Jaeger and Haas, 2004). TDNNs are feedforward NNs with delayed versions of inputs that implement a short-term memory mechanism (Waibel et al., 1989). Of these NNs, RNNs are highly accurate in BMI applications (Sanchez et al., 2004, 2005; Sussillo et al., 2012; Kifouche et al., 2014; Shah et al., 2019). Therefore, the present work designed an RNN with error feedback as the neural decoder.

Because the performance of an NN relied heavily on its network structure, structure selection is a crucial concern. An NN with an excessively large architecture may overfit the training data and yield poor generalization. Furthermore, it often exhibited rigid timing constraints. By contrast, an NN with an excessively small architecture may underfit the data and fail to approximate the underlying function. The four most frequently used algorithms to determine a network's architecture are constructive, pruning, constructive-pruning, and evolutionary algorithms (EAs). Constructive algorithms (Kwok and Yeung, 1997) began with a simple NN and then increased the number of hidden neurons or connections to that network in each iteration. However, an oversized network may be constructed due to inappropriate stopping criterion. In other words, the matter of when to stop constructing networks lacked consensus. The pruning algorithm (Reed, 1993) began with an oversized NN and then removed insignificant hidden neurons or connections iteratively. However, it was difficult to initially determine an oversized network architecture for a given problem (Kwok and Yeung, 1997). The constructive-pruning algorithm (Islam et al., 2009; Yang and Chen, 2012) combined both a constructive algorithm and pruning algorithm to build an NN. Starting with the simplest possible structures, the NN was first constructed using a constructive algorithm and then removed trivial hidden neurons or connections by using a pruning algorithm to achieve optimal network architecture. Several works have designed NNs using EAs (Huang and Du, 2008; Kaylani et al., 2009; Masutti and de Castro, 2009). EAs were developed as a biologically plausible strategy to adapt various parameters of NNs, such as weights and architectures (Angeline et al., 1994). However, encoding an NN into a chromosome depended on the maximum structure of the network, which is problem-dependent and must be defined by user. This property limited the flexibility of problem representation and the efficiency of EAs. One study (Yang and Chen, 2012) proposed an evolutionary constructive and pruning algorithm (ECPA) without predefining the maximum structure of the network, which made the evolution of the network structure more efficient. Because the NNs used in BMIs were designed in a subject-dependent manner, the automatic optimization of the NN for a specific task is a desired feature. This study adopted the ECPA (Yang and Chen, 2012) to develop an NN with an appropriate structure as a neural decoder for each subject in BMI applications.

This work proposed an evolutionary constructive and pruning neural network with error feedback (ECPNN-EF) to decode neural activity into the forelimb movement of a rat by using only a few days of data to train the neural decoder. A lever-pressing task for the rat was designed to evaluate the effectiveness of the proposed neural decoder. The error feedback providing the difference between the decoded and actual kinematics might compensate for decreases in decoding performance when the neural recording conditions change. Thus, the ECPNN-EF might achieve stable and accurate long-term decoding performance. The rest of this paper is organized as follows. First, we describe the experimental setup and the proposed decoder. Second, we demonstrate the influence of several parameters, namely the probabilities of *crossover* and *mutation*. Furthermore, the effects of evolution progress, cluster-based pruning (CBP) and age-based survival selection (ABSS) on the performance of the proposed decoder are also shown and discussed. Finally, we describe how partially connected topology and error feedback improve the long-term decoding performance.

## Materials and Methods

### Animals

Four male adult Wistar rats were aged 8 weeks old, weighed between 250 and 350 g, and were kept in the animal facility with well-controlled laboratory conditions (12: 12 light/dark cycle with light at 7 AM; 20° ± 3°C) and fed on *ad libitum*. The care and experimental manipulation of the animals were reviewed and approved by the Institutional Animal Care and Use Committee of the National Yang Ming University.

### Surgery for Neural Implantation

Animals were anesthetized with 40 mg/kg Zolazepam and Tiletamine (Zoletil 50, Virbac., Corros, France) and 8 μg/kg dexmedetomidine hydrochloride (Dexdomitor®, Pfizer Inc., New York, NY, USA) through intramuscular injections. Rats were positioned in a stereotaxic frame (Stoelting Co. Ltd., Wood Dale, IL, USA) and secured with the ear bars and tooth bar. An incision was made between the ears. The skin of the scalp was pulled back to expose the surface of the skull from the bregma to the lambdoid suture. Small burr holes were drilled into the skull for the microwire electrode array implanted and for the positioning of screws (Shoukin Industry Co., Ltd., New Taipei City, Taiwan).

For each rat, an 8-channel laboratory-made stainless microwire electrode array (product # M177390, diameter of 0.002 ft., California Fine Wire Co., Grover Beach, CA, USA; the electrodes were spaced 500 μm apart) was vertically implanted into the layer V of the forelimb territory of the primary motor (M1) cortex (anterior-posterior [AP]: +2.0 mm to −1.0 mm, medial-lateral [ML]: +2.7 mm, dorsal-ventral [DV]: 1.5 mm. For determining the location of the forelimb representation of M1 for the electrode implantation, the intracortical microstimulation was applied to confirm via forelimb muscle twitches observed (Yang et al., 2016). Following a 1-week post-surgery recovery period, the animals received the water reward-related lever-pressing training.

### Behavioral Training

The rats were trained to press a lever with their right forelimb to obtain a water reward. Before reward training, the rats were single-housed and deprived of water for at least 8 h. During reward training, the rats were placed in a 30 × 30 × 60 cm^3^ laboratory-designed Plexiglas testing box, and a 14 × 14 × 37.5 cm^3^ barrier was placed to construct an L-shaped path for the behavioral task. A lever (height of 15 cm from bottom) was set at one end of the path, and an automatic feeder with a water tube that provided water on a plate was set at the other end of the path. The rats could obtain 0.25-ml water drop as a reward on the plate after pressing the lever. Thirsty rats were trained to press a lever in order to receive water reward without any cues because they learned to make an operant response for positive reinforcement (water reward). Rats were trained to press a lever on the left side of the box then freely move along the U-shaped path to the right side of the box. This had to be completed within 3 s to receive a reward. The experimental time course included the behavioral training and data collection phases. In the behavioral training phase, implanted rats learned the lever pressing and water reward association within 3–5 d without neural recordings. To meet criteria for successful learning of the behavioral task, the rats had to complete continuous repetition of five successive trials of associated lever pressing and water reward without missing any trial between successive trials (Lin et al., 2016). Once reaching the criteria, animals entered the data collection phase. During this 12-d phase, forelimb movement trajectories were simultaneously acquired with corresponding electrophysiological recordings of neural spikes as they performed the water reward task.

### Data Recording

**Figure 1:**
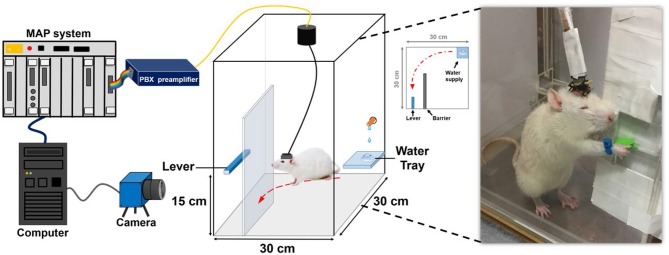
System architecture and experimental setting of the water-reward lever-pressing task. While the rat was pressing the lever to obtain the water reward, the neural recording system recorded and preprocessed the neuronal spiking activity from the electrode array implanted in the rat's cortex. The trajectory-tracking system acquired the corresponding forelimb trajectory from the camera.

In this study, forelimb kinematics and neuronal activity were simultaneously recorded while the animal performed the water reward-related lever pressing as shown in Figure 1. During the behavioral task, a blue-colored marker made of nylon was mounted on the right wrist of the rat to track forelimb trajectory. The trajectory of the rat's forelimb movement was captured by a charge-coupled device camera (DFK21F04, Imaging Source, Bremen, Germany) that provided a 640 × 480 RGB image at 30 Hz and then analyzed by a video tracking system (CinePlex, Plexon Inc., Dallas, TX, USA). When the lever was pressed, it triggered the micro-switch of the pull position to generate a transistor–transistor logic pulse to the multichannel acquisition processor (Plexon Inc., Dallas, TX, USA) which allowed the neuronal data to be accurately synchronized to the lever pressing event and then water reward was delivered through a computer-controlled solenoid valve connected to the laboratory-designed pressurized water supply.

Neuronal spiking activity of the rat was sampled at 40 kHz and analog filtered from 300 to 5,000 Hz. A spike-sorting algorithm was used to determine single-unit activity. First, an amplitude threshold with four standard deviations of filtered neuronal signals was set to identify spikes from the filtered neuronal signals. Then, spikes were sorted by a trained technician through principal component analysis using a commercial spike-sorting software (Sort Client, Plexon Inc., Dallas, TX, USA).

### Neural Decoder: ECPNN-EF

**Figure 2:**
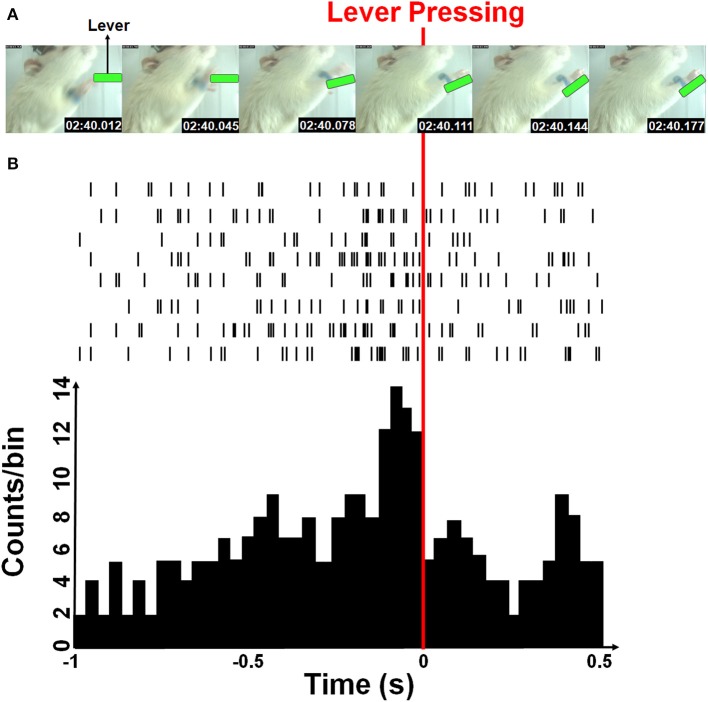
Simultaneous forelimb movement trajectory and spike train recordings during the water-reward-related lever-pressing task. **(A)** Stop-motion animation representing forelimb movement from the video-tracked time-series data (see Supplemental Video). Six consecutive photographs showed a rat in the test cage successfully reaching and pressing the lever (marked with green) while the forelimb movement trajectories and neural activity were simultaneously recorded. **(B)** Neuronal activities recorded from eight neurons during one movement displayed as spike trains and the neuronal activity histogram (a bin size of 33 ms). The red line indicates the moment when the rat pressed down the lever with its right forelimb.

The firing rates of each sorted unit from M1 cortex of rat were decoded into the instantaneous velocity of the forelimb trajectory. Both horizontal and vertical velocities were estimated from the position of the blue-colored marker by a two-point digital differentiation. The firing rates of each sorted neuron was determined by counting spikes in a given time bin whose length was 33 ms and was equal to the temporal resolution of the video tracking system. Figure 2 showed an example of the rat forelimb movement while pressing the lever and corresponding neural spike trains. A time-lag was known to exist between neuronal firing and the associated forelimb state because of their causal relationship (Paninski et al., 2004; Wu et al., 2004; Yang et al., 2016). Furthermore, the decoding accuracy was improved when the optimal time-lag is considered. Here, the water-restricted rats easily learn a lever pressing behavior within a few training sessions, allowing for recording neuronal activity during acquisition of a motor sequence as shown in Figures 2A,B showed some M1 neurons displayed increased activity for sequential motor behavior prior to the lever-pressing event, which presented the maximum firing rate at the third time-bin (with 99 ms lag). Therefore, we empirically choose 363 ms of spike train over 11 time-bins (8 bins before and 2 bins after the 3rd time-bin prior to the lever pressing) to predict a series of movement velocities.

**Figure 3:**
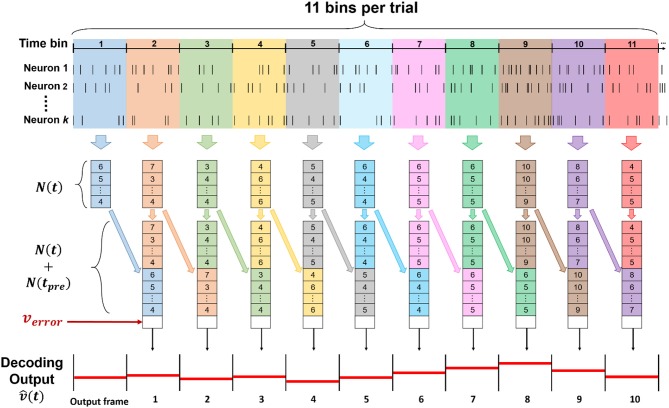
The data structure of input for the ECPNN-EF decoder. The input of the decoder was consisted of (*t*), *N*(*t*_*pre*_) and *v*_*error*_, whose length was 2*k* + 1, including 2*k* spike counts of both concurrent and preceding bin, and an error feedback calculated by $\left|v\left(t_{pre}\right)-\hat{v}\left(t_{pre}\right)\right|$. To predict a whole forelimb movement in a trial, 11 bins were used and decoded to $\hat{v}\left(t\right)$ in the time series.

The spike train was discretized in 11 time-bins for each trial, corresponding to each entire trajectory of forelimb movements during the lever reaching task. With total *k* neurons sorted from all channels, we defined $N\left(t\right)={\left\{n_{i}\left(t\right)\right\}}_{i=1}^{k}$ as a set of neuronal features, where *t* denoted time step which was time bin in this study, *n*_*i*_(*t*) represented as spike count of sorted neuron in current bin, and *i* denoted index of sorted neuron from 1 to *k*. In this study, we used both concurrent and preceding bin as neuronal features, which is *N*(*t*) and *N*(*t*_*pre*_), to predict current velocity $\hat{v}\left(t\right)$, where *t* and *t*_*pre*_ represented current time bin and preceding time bin, respectively. The step of data processing was shown in Figure 3.

The ECPNN-EF is an RNN-based neural decoder designed using the ECPA as proposed in Yang and Chen (2012). The input–output function of the ECPNN-EF was denoted by:

(1) $$\begin{array}{l} \hat{v}\left(t\right)=f_{W}\left(N\left(t\right),N\left(t_{pre}\right),v_{error}\right) \end{array}$$

where $\hat{v}\left(t\right)$ represented the predicted velocity and *W* represented the weights of the ECPNN-EF. The prediction error *v*_*error*_ was adopted as the error feedback which was the absolute value of difference between the actual velocity and predicted velocity, and was calculated by $v_{error}=|v\left(t_{pre}\right)-\hat{v}\left(t_{pre}\right)|$ where *t*_*pre*_ denoted as preceding time bin.

**Figure 4:**
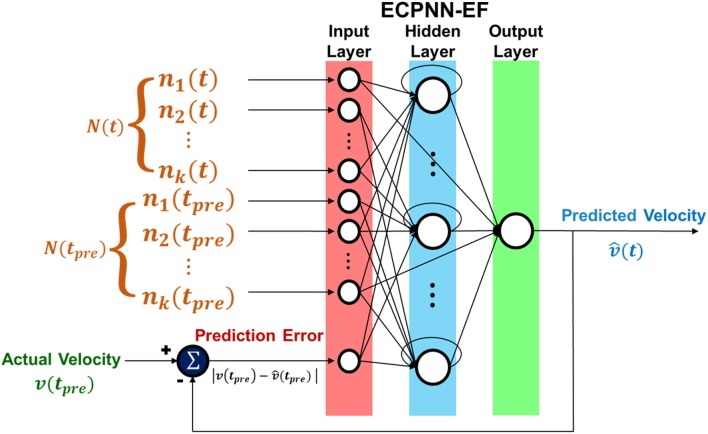
Structure of ECPNN-EF. Network included input, hidden, and output layer. ECPNN-EF took the neuronal feature from combination of concurrent bin *N*(*t*), preceding bin *N*(*t*_*pre*_) and prediction error (error feedback) *v*_*error*_ to predict velocity $\hat{v}\left(t\right)$.

The structure of ECPNN-EF was showed in Figure 4. Note that there was only one output neuron representing the predicted velocity in the neural decoder. The vertical and horizontal velocities were predicted in two separate neural decoders.

Mean squared error was adopted as loss function due to its wide use in regression application and was defined as follows:

(2) $$\begin{array}{l} L=\underset{t}{\sum }{\left(v_{t}-f_{W}\left(N\left(t\right),N\left(t_{pre}\right),v_{error}\right)\right)}^{2} \end{array}$$

The optimal weights of the ECPNN-EF were obtained by minimizing the loss as follows:

(3) $$W^{*}=\text{arg }\underset{W}{\text{min}}\;L$$

This study applied backpropagation through time (BPTT) to find the optimal weights of the ECPNN-EF by iteratively determining the gradient of the loss with respect to the weights as follows:

(4) $$\begin{array}{l} W\leftarrow W-\eta \nabla_{W}L \end{array}$$

where η is learning rate. Details of the BPTT (Werbos, 1988) are described in the Supplementary Note 1. The details of designing structure of the ECPNN-EF are described in the Supplementary Note 2.

The ECPNN-EF adopted a hyperbolic tangent sigmoid transfer function for all hidden neurons and the output neuron. Skip connections existed between discontinuous layers, such as from the input layer to the output layer. Furthermore, the hidden layer possessed self-recurrent connections. After both the structure and weights of the ECPNN-EF had been trained, the fixed model was adopted to predict the velocity of the rat's forelimb without the additional cost of training.

The pseudo code of ECPNN-EF training algorithm appears in Algorithm 1. The initial population started with a set of initial NNs, each NN of which had a single hidden neuron. A single connection was generated from one non-error-related input neuron to the hidden neuron. A skip connection was generated from one non-error-related input neuron to the output neuron. A single connection was generated from one error-related input neuron to the hidden neuron or to the output neuron. The detail description of population initialization was in the Supplementary Note 3. Furthermore, a self-recurrent connection was constructed in the hidden neuron with a probability of 0.5. Here, the error-related input neuron received the prediction error, as indicated in Figure 4, whereas non-error-related input neurons received the instantaneous firing rate of each unit. This mechanism of separately generating connections of error-related input neuron and non-error-related input neurons ensures that the initial NNs can immediately process error feedback. As a result, a set of initial NNs with partially connected topology was generated.

The purpose of the *network crossover operator* was to explore the structural search space and thus improve the processing capabilities of the ECPNN-EF. The *network crossover operation* randomly selected two parent NNs through tournament selection and then combined their structures to generate an offspring NN with a *crossover probability*, *p*_*c*_ (see Supplementary Note 4). The *network mutation operator* exploited the structural search space to achieve a small perturbation of structure by randomly generating a new connection from the input to the hidden neuron. Furthermore, the *network mutation operation* randomly constructed a self-recurrent connection of a hidden neuron or a new skip connection from the output or the hidden neuron to its previous consecutive or non-consecutive layer with a *mutation probability*, *p*_*m*_ (see Supplementary Note 5).

CBP mainly pruned insignificant hidden neurons to avoid an excessively complex ECPNN-EF with poor generalization performance owing to the use of *network crossover operation*. It first clustered the hidden neurons into two groups (i.e., better and worse groups, depending on their significance in the NN) and then removed the hidden neurons in the worse group in a stochastic manner. The detailed description of CBP was in the Supplementary Note 6.

ABSS prevented the ECPNN-EF from achieving a fully connected structure. It selected NNs for the next generation according to age, which indicated how many generations the NN had survived. Older NNs tended to have a fully connected structure because of the use of *network mutation operation*. ABSS replaced old age NNs with initial NNs (see Supplementary Note 3) in a stochastic manner and thus prevented the population from achieving a fully connected structure. ABSS mainly removed fully connected networks and made the rest of the NNs survive to the next generation through a stochastic mechanism. The detailed description of ABSS was in the Supplementary Note 7. The evolution process terminated when a generalization loss (*GL*) met an early stopping criterion or the maximum number of generations was reached. The early stopping criterion motivated from Islam et al. (2009) evaluated the evolution progress using training and validation errors in order to avoid overfitting. It first defined the *GL* at the τth generation as:

(5) $$\begin{array}{l} GL\left(\tau \right)=\frac{E_{va}\left(\tau \right)}{E_{low}\left(\tau \right)}-1 \end{array}$$

where *E*_*va*_(τ) is a validation error of the NN with the best fitness at the τth generation and *E*_*low*_(τ) is the lowest *E*_*va*_(τ) up to the τth generation. The difference between the average training error and the minimum training error at the τth generation of a strip *k* was defined as:

(6) $$\begin{array}{l} P_{k}\left(\tau \right)=\frac{\sum_{\omega =\tau -k+1}^{\tau }E_{tr}\left(\omega \right)}{k\times \min_{\omega =\tau -k+1}^{\tau }E_{tr}\left(\omega \right)}-1 \end{array}$$

where *E*_*tr*_(ω) is the training error of the NN with the best fitness at the ωth generation and *k* is the strip length. *k* was set to 5 in this work. Note that *GL*(τ) and *P*_*k*_(τ) were determined using the validation and training sets, respectively. *E*_*va*_(τ) and *E*_*tr*_(ω) were calculated by the loss function provided in **(2)**. The ECPNN-EF training algorithm terminated when *GL*(τ) > *P*_*k*_(τ). The optimal NN with a partially connected topology was selected as the neural decoder.

In summary, *network crossover* and *mutation* evolved NNs in a constructive manner to improve their processing ability, whereas CBP and ABSS evolved NNs in a destructive way that enhanced their generalization capabilities and reduced hardware costs (Yang and Chen, 2012). An early stopping criterion was adopted to terminate the evolution process by observing both training and validation errors to avoid overfitting during training phase, which reduced the training time and retained generalization capability (Islam et al., 2009). The ECPNN-EF was implemented and trained in MATLAB (MathWorks, Natick, MA, USA).

### Data Sets and Optimizing the Structure of Neural Networks

Data collected in a recording session were divided into a training set for developing the neural decoder, a validation set for avoiding overfitting during training phase, and a testing set for evaluating the generalization ability of the neural decoder. For each rat, the experimental trials of the first 2 days were used as training and validation sets, and the remaining 10 days were used as testing set. The number of trials used for each rat was shown in Table 1.

**Table 1:** Experimental data characteristics.

**Animal no**.	**Number of trials for training in day 1 and day 2**	**Number of trials for testing per day**	**Number of neurons used per trial**
Rat #11	70	24	8
Rat #14	80	33	12
Rat #16	140	41	8
Rat #17	95	36	8

*Experimental data recorded from four rats were used in this study. The decoder of each rat was trained and tested only by its own experimental trials. The experimental data of the first 2 days and subsequent 10 days were used for training and testing, respectively. The criteria for selecting neurons into neural decoder as the input were described in the Supplementary Note 8*.

The present study evaluated the prediction accuracy (decoding performance) of the proposed neural decoder using Pearson's correlation coefficient (***r***), which measured the strength of a linear relationship between the observed and predicted forelimb trajectories (Manohar et al., 2012; Shimoda et al., 2012). When evolving ECPNN-EF with good generalization ability and compact structure, a 5-fold cross validation was adopted to determine the optimal *p*_*c*_, *p*_*m*_ and terminated generation. For each given pair of *p*_*c*_ and *p*_*m*_, the experimental trials of the first 2 days were randomly partitioned into five equal-sized disjoint sets where four sets were used as the training set to evolve ECPNN-EF and one set was used as the validation set to evaluate the decoding performance of the evolved ECPNN-EF during training phase. Once the ECPNN-EF was evolved through the optimal *p*_*c*_ and *p*_*m*_, the validation set also was used to determine the best terminated generation of the evolved ECPNN-EF.

To investigate the effects of CBP and ABSS on the ECPNN-EF evolution, two variants of ECPNN-EF that only adopted either CBP or ABSS were implemented. One variant of ECPNN-EF only with CBP was referred to as ECPNN-EFWC, and the other variant of ECPNN-EF only with ABSS was referred to as ECPNN-EFWA. The decoding performances of ECPNN-EF, ECPNN-EFWC, and ECPNN-EFWA were compared using the validation set in terms of ***r***, number of hidden neurons (*N*_*h*_), number of connections (*N*_*c*_), connection ratio (*R*_*c*_), and termination generation (*G*_*T*_). The *R*_*c*_ was defined as follows:

(7) $$\begin{array}{l} R_{c}=\frac{N_{c}}{N_{f}} \end{array}$$

where *N*_*f*_ is the number of connections in a network with a fully connected topology. The network had fully connected topology when *R*_*c*_ = 1; however, the network had partially connected topology when *R*_*c*_ < 1.

### Statistical Analysis

In this study, we investigated the decoding performance dependency on the parameters of *p*_*c*_ and *p*_*m*_by employing the statistical method, two-way analysis of variance (ANOVA) followed by Tukey's *post-hoc* test and adjusted the *P*-value by multiple comparison using Bonferroni correction, on the validation set. We set *p*_*c*_ at 7 levels (0.6, 0.65, 0.7, 0.75, 0.8, 0.85, and 0.9). In addition, 7 levels of *p*_*m*_ (0.6, 0.65, 0.7, 0.75, 0.8, 0.85, and 0.9) were employed for each *p*_*c*_ to determine whether the algorithm found the near-optimum solution. This evaluated whether there were any significant differences in decoding performance according to the parameters used. Additionally, we analyzed the effects of CBP and ABSS on the ECPNN-EF reconfiguration, and then assessed the decoding performance comparison of ECPNN-EF, ECPNN-EFWC, and ECPNN-EFWA on the validation set by one-way ANOVA with *post-hoc* Tukey's HSD test.

In order to investigate the decoding performance and stability of ECPNN-EF as well as impact of the prediction error feedback on enhancing the prediction accuracy of ECPNN-EF without error-correction learning (ECPNN), a mixed model ANOVA with three decoders [ECPNN-EF, a fully connected RNN with error feedback (RNN-EF), and ECPNN] as fixed factors and the repeated measure of daily testing set over 10 testing days followed by Tukey's *post-hoc* test and then adjusted the *P*-value by Bonferroni multiple comparison correction. The decoding performances of the four rats were presented as means ± standard deviation (SD). The data analysis was performed in SPSS version 20.0 (SPSS Inc., Chicago, IL, USA).

## Results

### On the Decoding Performance of Different Crossover and Mutation Probabilities

**Figure 5:**
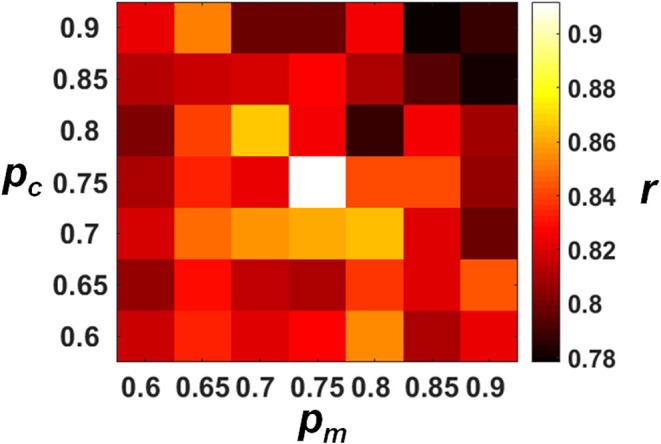
Decoding performance of the ECPNN-EF under various *p*_*c*_ and *p*_*m*_. We performed the *post-hoc* analysis based on the estimated marginal means of correlation coefficient (***r***) and adjusted the *P*-value by multiple comparison using Bonferroni correction (see the Table S2 in the Supplementary Note 8). We found that the highest ***r*** was observed when the *p*_*m*_= 0.75 and the *p*_*c*_ = 0.75 (***r*** = 0.912 ± 0.019) and showed significant differences in ***r*** of *p*_*c*_ = 0.8 and *p*_*m*_ = 0.75. Therefore, the near-optimum solution of the algorithm was *p*_*c*_= 0.75 and *p*_*m*_ = 0.75.

The decoding performances of the ECPNN-EF evolved under different *p*_*c*_ and *p*_*m*_ values were evaluated by the validation set as shown in Figure 5. The results demonstrated that the more increasing in *p*_*c*_ and *p*_*m*_ and the worse decoding accuracy (***r***). A simple main effects analysis which examined the effects of 7 levels of *p*_*m*_ at the fixed level of *p*_*c*_ = 0.75 and 0.8 was provided in the Supplementary Note 9. The best decoding performance (***r*** = 0.912 ± 0.019) was achieved using *p*_*c*_ = 0.75 and *p*_*m*_ = 0.75 (compared to other combinations of *p*_*c*_ and *p*_*m*_, *P* < 0.05 analyzed by ANOVA for multiple comparisons).

### Evolution Progress of ECPNN-EF

**Figure 6:**
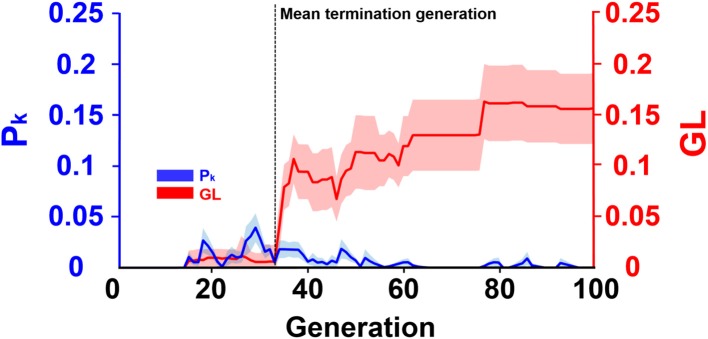
Evolution progress of the ECPNN-EF. The shaded regions represented SD. The vertical axis on the left represented *P*_*k*_ (blue line). The vertical axis on the right represented *GL* (red line). Most *GL*s met the early stopping criterion in the mean terminated generation (*G*_*T*_ = 33.2 ± 1.1) indicated by a black vertical dashed line.

Figure 6 presented the evolution progress of the ECPNN-EF using the optimal *probabilities* of *crossover* and *mutation* (*p*_*c*_ = 0.75 and *p*_*m*_ = 0.75) obtained in Figure 5. The results showed that the *GL* and the difference between the average training error and minimum training error (*P*_*k*_) were almost zero in the early generations. Afterward, the *GL* slightly increased but the *P*_*k*_ varied. Notably, the *GL* was not consistently larger than the *P*_*k*_. Most *GL*s dramatically increased and were larger than the *P*_*k*_ after the 33th generation marked by a black vertical dashed line. This potentially indicated the overfitting problem that might lead to worse evolutions. Therefore, the ECPNN-EF was suggested to terminate evolution in this generation according to an early stopping criterion in order to maintain stable decoding performance. The mean termination generation was 33 in this study (*G*_*T*_ = 33.2 ± 1.1).

### Effects of CBP and ABSS on Decoding Performance

Table 2 showed the decoding performance of the ECPNN-EF, ECPNN-EFWC, and ECPNN-EFWA. ECPNN-EF achieved significantly higher decoding performance (***r*** = 0.912 ± 0.019) than did the ECPNN-EFWC (***r*** = 0.602 ± 0.083) and ECPNN-EFWA (***r*** = 0.708 ± 0.066) (*P* < 0.05 analyzed by one-way ANOVA with *post-hoc* Tukey's HSD test). The ECPNN-EF possessed a more compact structure (*N*_*h*_ = 4.2 ± 2.7 and *N*_*c*_ = 20.6 ± 7.2) than both ECPNN-EFWC (*N*_*h*_ = 4.6 ± 3.8 and *N*_*c*_ = 23.0 ± 10.1) and ECPNN-EFWA (*N*_*h*_ = 14.4 ± 14.4 and *N*_*c*_ = 57.9 ± 52.1). Moreover, the ECPNN-EFWA had a greater standard deviation than the other two methods. All three methods possessed almost the same *R*_*c*_. The ECPNN-EF (*R*_*c*_ = 0.12 ± 0.02) and ECPNN-EFWA (*R*_*c*_ = 0.12 ± 0.01) produced slightly more sparse structures than ECPNN-EFWC (*R*_*c*_ = 0.13 ± 0.01). All three methods terminated the evolution before 38th generation. The ECPNN-EF terminated slightly earlier (*G*_*T*_ = 33.2 ± 1.1) than did the other two decoders.

**Table 2:** Evolutionary results of ECPNN-EF, ECPNN-EFWC, and ECPNN-EFWA.

**Decoder**	***R***	***N*_*h*_**	***N*_*c*_**	***R*_*c*_**	***G*_*T*_**
ECPNN-EF	0.912 ± 0.019^*^	4.2 ± 2.7	20.6 ± 7.2	0.12 ± 0.02	33.2 ± 1.1
ECPNN-EFWC	0.602 ± 0.083	4.6 ± 3.8	23.0 ± 10.1	0.13 ± 0.01	37.5 ± 7.9
ECPNN-EFWA	0.708 ± 0.066	14.4 ± 14.4^*^	57.9 ± 52.1^*^	0.12 ± 0.01	37.3 ± 3.5

The symbol ^*^indicated statistical significance among the three decoders (P < 0.05 analyzed by one-way ANOVA with post-hoc Tukey's HSD test).

### Decoding Performance Comparison

**Figure 7:**
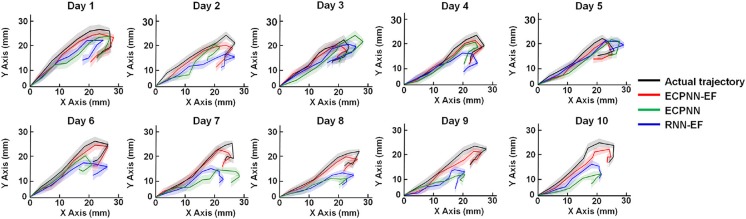
Data visualization of average predicted trajectories of the ECPNN-EF, ECPNN, and RNN-EF. Representative daily reconstructed trajectories of the test trials in Rat #16. The average reconstructed trajectories of the ECPNN-EF (red line) were more similar to the actual ones (black line) and exhibited less variance than did those of the ECPNN (green line) and RNN-EF (blue line) over 10 test days, where shadow regions represented their corresponding SDs of the predicted trajectories.

We applied a mixed model ANOVA with three decoders (ECPNN-EF, RNN-EF, and ECPNN) as fixed factors and the repeated measure of time, then adjusted the *P*-value by multiple comparison using Bonferroni correction. The three decoders reconstructed movement trajectories similar to the actual movement trajectories (Figure 7). However, the ECPNN-EF decoder showed the best reconstruction and stability and was significantly better than the ECPNN and RNN-EF decoders over 10 test days.

**Figure 8:**
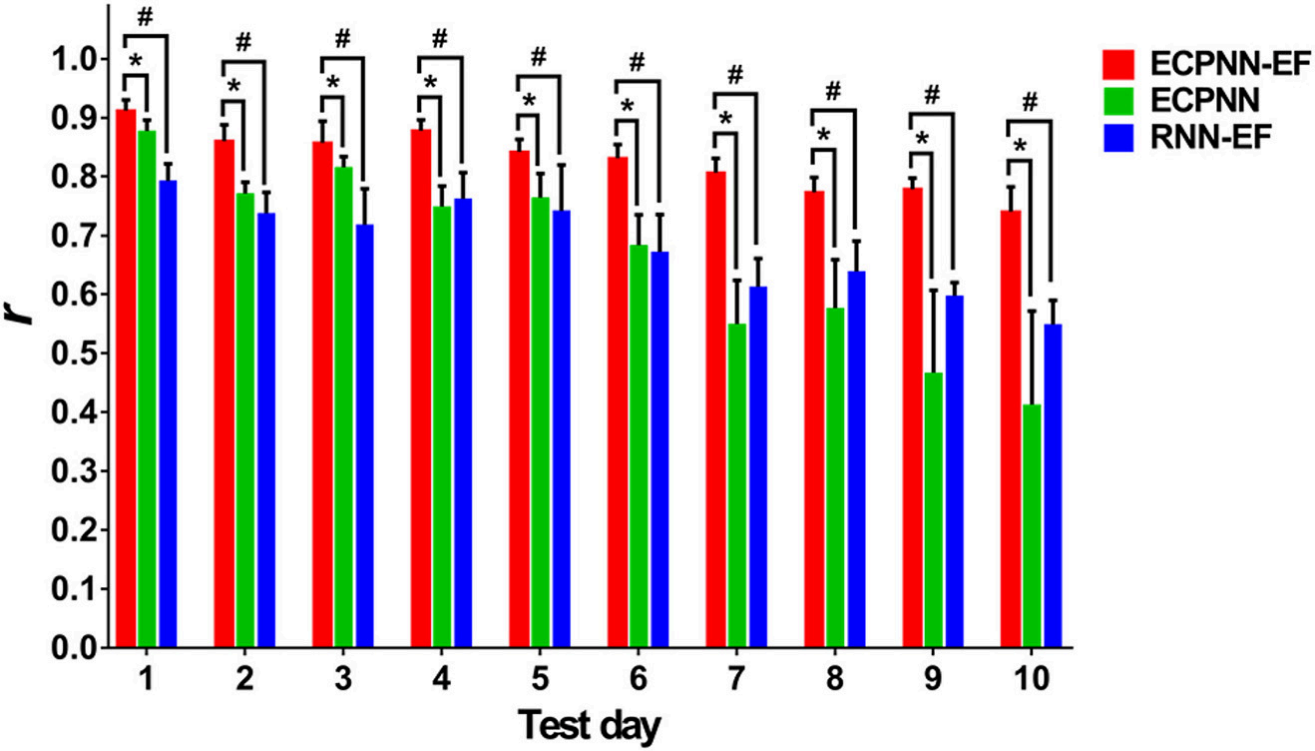
Comparison of daily *r* of the ECPNN-EF, ECPNN, and RNN-EF. The decoding performance of ECPNN-EF at the 1st, 2nd, 3rd, 4th, 5th, 6th, 7th, 8th, 9th, and 10th. Test Day was significantly higher than those of ECPNN and RNN-EF (also see the *post-hoc* analysis of the comparison of decoding performance in the Table S3 in the Supplementary Note 10), and the corresponding variation of ***r*** was smaller than that of the ECPNN and RNN-EF after Test Day 4. The symbols * and ^#^ indicate *P* < 0.05, as analyzed by the repeated measures analysis using mixed model ANOVA with Bonferroni correction for multiple testing.

To investigate the effectiveness of the partially connected topology of ECPNN-EF, the ECPNN-EF was compared with RNN-EF using the *post-hoc* analysis. Here, the number of hidden neurons of the RNN-EF was the same as that in the ECPNN-EF for fair comparison [the weights were adjusted by the BPTT] (Werbos, 1988). Figure 8 statistically showed the daily ***r*** comparison between the decoders of ECPNN-EF and RNN-EF. The mean ***r*** of the decoder of RNN-EF monotonically decreased with gradually increasing in the variability of ***r*** over 10 test days. The result showed that the RNN-EF could not offer a stable long-term decoding performance. By contrast, the decoding performance of the ECPNN-EF decreased slightly in each day and achieved ***r*** = 0.740 ± 0.042 at Test Day 10. Moreover, the variation in neural decoding performance (SD) of the ECPNN-EF was smaller than that of the RNN-EF in each day. The decoding performance of the ECPNN-EF was significantly higher than that of RNN-EF (*P* < 0.05 analyzed by repeated measures analysis using mixed model ANOVA with *post-hoc* test, *N* = 4) in each day.

To investigate the effect of the error-correction learning (error feedback) in the decoder, the ECPNN-EF was compared with ECPNN using the *post-hoc* test. As depicted in Figure 8, the ECPNN-EF decoder performed higher and more stable accuracy of predicted trajectories in comparison with those of the ECPNN decoder over 10 test days. The mean ***r*** of the decoder of ECPNN dropped noticeably and the corresponding variability of ***r*** became huge after Test Day 5. By contrast, the ECPNN-EF's daily ***r*** slowly decreased, and the daily variability of ***r*** did not considerably change over 10 days. The ECPNN-EF's ***r*** was significantly higher than that of the ECPNN in each day (*P* < 0.05 analyzed by repeated measures analysis using mixed model ANOVA with *post-hoc* test, *N* = 4), and the corresponding variation (SD) in daily ***r*** of the ECPNN-EF was smaller than that of the ECPNN. The lowest ***r*** of the ECPNN-EF (***r*** = 0.740 ± 0.042) and ECPNN (***r*** = 0.413 ± 0.158) was observed at Test Day 10.

## Discussion

### Best Performance With NN Structure Determined by Near Optimal Probabilities of Crossover and Mutation

The *p*_*c*_ and *p*_*m*_ affected the evolution of the network structure and thus involved neuronal contributions to forelimb movement. Both *crossover* and *mutation operators* increased the model complexity. The *crossover* provided a chance to add hidden neurons while the *mutation* achieved a small perturbation of model structure by adding connections. Previous paper Schwartz et al. (1988) has reported that individual neuron in the motor cortex discharges with movements in its preferred direction. A high *p*_*c*_ led to large network structure which possessed sufficient information processing capability but might result in overfitting. On the contrary, a low *p*_*c*_ led to simple network structure which might result in underfitting. A high *p*_*m*_ allowed the hidden neurons to have more connections from the neuronal inputs which led to fully connected topology. Increasing *p*_*c*_ and *p*_*m*_ may not consistently improve the decoding performance of the evolved neural decoder. Increasing *p*_*c*_ and *p*_*m*_ from 0.6 to 0.75 enlarged the computational complexity of the neural decoder so that relevant neuronal inputs could be accurately decoded into forelimb movement. However, frequent *crossover* or *mutation* (high *p*_*c*_ and *p*_*m*_, respectively) in the 0.8–0.9 range may introduce redundant connections from irrelevant neurons with firing rates that did not contribute to the kinematic parameters. Conversely, a low *p*_*m*_ allowed the hidden neurons to have few connections, resulting in sparse topology. However, some neuronal inputs had a lower likelihood of being processed. Low *p*_*c*_ and *p*_*m*_ may reproduce a topology that is too sparse to build connections between kinematic parameters and relevant neurons, resulting in less accurate neural decoding. Our experimental results showed that *p*_*c*_ = 0.75 and *p*_*m*_ = 0.75 could achieve the best decoding performance. The evolved ECPNN-EF possessed not only sufficient hidden neurons to decode neuronal activities, but appropriate topology which selected forelimb movement related inputs to the hidden neurons.

### Early Stopping to Counteract Overfitting in Evolutionary Progress of ECPNN-EF

Most evolutions of the ECPNN-EF terminated around the mean termination generation because the early stopping criterion was met. The early stopping criterion employed both training and validation errors. In the early generations, the *GL* was almost zero, which indicated that the validation error was almost the same with the lowest validation error among the recent generations. This indicates that the validation error did not increase. Although the functional mapping between neural activity and kinematic parameters varied across days due to variability in the neural recording conditions, the training set may have similar neural recording conditions as the validation set. The ECPNN-EF learned the common functional mapping of the training and validation sets in the early generations, allowing for its evolved sparse topology to gradually learn to decode common firing patterns into forelimb movements in the validation set. Furthermore, the *P*_*k*_ was almost zero, which indicated that the average training error was not larger than the minimum training error among the recent generations. This demonstrated that the training error gradually decreased. Both the *GL* and *P*_*k*_ indicated that the evolution improved the generalization ability of the ECPNN-EF in the early generations. Before the mean termination generation, the slight increase of the *GL* might indicate overfitting, but the *GL* was not always higher than the *P*_*k*_. This implied that the generalization ability of the ECPNN-EF had a chance to be repaired by the evolution as illustrated in Prechelt (1998); Sussillo et al. (2016). Most *GL*s dramatically increased and were consistently higher than the *P*_*k*_ after the mean termination generation. This demonstrated that the validation error increased, indicating overfitting. Previous work (Kao et al., 2015) has suggested that a neural decoder with too many parameters may result in overfitting. The evolution tended to construct a more complex neural decoder with several weights, potentially contributing to overfitting in the later generations. Therefore, the evolution terminated to prevent decreased generalization ability from overfitting the training set and to save computational time.

### Best Performance Based on NN With Good Generalization Ability and Compact Structure

The fact that the ECPNN-EF significantly outperformed the ECPNN-EFWC and ECPNN-EFWA in terms of ***r*** suggested that both CBP and ABSS were essential to evolve the neural decoder with generalization ability. The CBP pruned insignificant hidden neurons and led to lower *N*_*h*_ and *N*_*c*_ in ECPNN-EF and ECPNN-EFWC. This mechanism made the network more compact and prevented the network from excessively complex structure caused by the network crossover through many generations. On the other hand, the ECPNN-EFWC's *R*_*c*_ was expected to be considerably larger than those of ECPNN-EF and ECPNN-EFWA because the ABSS tended to select network with sparsely connected topology. However, the difference of *R*_*c*_ among the ECPNN-EF, ECPNN-EFWC, and ECPNN-EFWA was not significant because of the effect of early stopping. All the three approaches stopped evolution before 38 generations. The networks in the population underwent only few *crossovers* and *mutations*, and thus their network structures were less complex. Nevertheless, the poor ***r*** in the ECPNN-EFWC suggested that although early stopping led to lower *R*_*c*_, the evolution without ABSS would evolve a neural decoder with poor generalization ability. ABSS selected networks without redundant connections into next generation and thus prevented the network from fully connected topology caused by the network mutation. Excessively complex neural decoders may include redundant hidden neurons that overfit the training set. This can disrupt accurate decoding of neural activity in the testing set, which may have different neural recording conditions from the training set. Furthermore, redundant weights may connect to neurons with preferred directions that are irrelevant to vertical or horizontal velocities.

The ECPNN-EF terminated and obtained near-optimum neural decoder earlier than the ECPNN-EFWC and ECPNN-EFWA. The validation error in these three models increased after their termination generation. This resulted in overfitting because their evolved structures were more complex than the near-optimum neural decoders. Evolution of the ECPNN-EF was more efficient than the ECPNN-EFWC and ECPNN-EFWA, indicating that it obtained near-optimum neural decoder faster than its reduced models. Thus, ECPNN-EF's termination generation was earlier than its reduced models. These results indicate that CBP and ABSS helped evolve a neural decoder with less complex structure and better generalization ability. Power efficiency and power management are extremely important concerns for fully implantable neural decoders in BMIs. Due to its sparse topology, ECPNN-EF offers a practical approach to computationally efficient neural decoding by reducing the number of hidden neurons and interlayer connections, resulting in less memory usage and power consumption (Chen et al., 2015). Less power consumption results in a longer battery lifetime, which could facilitate brain implantation of neural decoders (Sarpeshkar et al., 2008).

### Best Performance Based on Appropriate Connected Topology-Based Network

Several studies have shown that a partially connected NN (PCNN) achieves better performance than does a fully connected neural network (FCNN) (Elizondo and Fiesler, 1997). The ECPNN-EF, a type of PCNN, achieved significantly higher daily mean ***r*** than does the RNN-EF, which is a type of FCNN; this suggests that an FCNN might consist of a large amount of redundant connections and lead to overfitting with poor generalization when compared to a PCNN (Elizondo and Fiesler, 1997; Wong et al., 2010; Guo et al., 2012). Some information, which was irrelevant to the forelimb movement and was processed by the redundant connections, may hamper the performance of the neural decoder and increase the likelihood of NNs being stuck in local minima. Furthermore, the variation in decoding performance of the RNN-EF from Test Day 3 to Test Day 10 was larger than that for Test Day 1 and Test Day 2, whereas the variation in decoding performance of the ECPNN-EF did not change dramatically. This suggested that the redundant weights in the RNN-EF could not deal with the variation of the neural recording conditions and thus led to unstable decoding performance. The ECPNN-EF outperformed the RNN-EF due to the use of the partially connected topology. The trends observed in the present study followed the suggestion that the number of connections is not the key aspect of an NN but rather of an appropriate connected topology (Yang and Chen, 2012).

### Comparing Linear Neural Decoder-Based Error-Correction Learning

Our prior work demonstrated a linear decoding model of the relationship between neural firing and kinematic parameters (Yang et al., 2016). A sliced inverse regression (SIR) with error-feedback learning (SIR-EF) was implemented based on an SIR linear neural decoder to fairly compare to the ECPNN-EF algorithm (see Supplementary Note 11). Because the ECPNN-EF had to process changing neural recording conditions over time, it possessed more processing capabilities than the linear model. The SIR-EF could not deal with long-term variability in neural recording conditions because of linear properties and limited computational complexity. The SIR-EF assigned weights to the slices with neurons that had a similar contribution to the lever-pressing forelimb movement. However, variations in neural recording conditions due to the tissue's reaction to neural implants or micromotion of the electrodes across days resulted in firing pattern variations (Barrese et al., 2013; Sussillo et al., 2016). Thus, the decoding performance decreased because the weights calculated using the training data over the first 2 days could not predict velocity in the subsequent testing days with different neural conditions.

### NN-Based Error-Correction Learning to Improve Long-Term Decoding Stability

It has been revealed that the functional mapping between instantaneous firing rate and kinematic parameters might vary in chronic recording due to the changes of neural recording conditions (Sussillo et al., 2016). A relative increase in ECPNN's mean ***r*** at Test Day 3 and Test Day 5 might exhibit that the recording conditions probably had some commonality with those in the training phase. Therefore, the non-linear model of ECPNN, which learned the time-dependent functional mapping from the training set, could accurately decode the instantaneous firing rate into kinematic parameters. The error feedback played a subsidiary role of the neural decoding in this situation. A considerable decrease in ECPNN's mean ***r*** after Test Day 5 might indicate that the neural recording conditions were different from those in the training phase. The learned functional mapping between instantaneous firing rate and kinematic parameters was of no use for making ECPNN's long-term decoding performance stable.

In contrast, when the neural recording conditions changed after Test Day 5, ECPNN-EF′ error feedback provided immediate kinematic information and thus compensated for across-day changes in functional mapping between instantaneous firing rate and kinematic parameters. The ECPNN-EF could achieve not only more robust within-day decoding (smaller SD) but also more robust across-day decoding (smaller fluctuations in daily mean ***r***) than those of the ECPNN. This demonstrated that employing error feedback in the ECPNN-EF improved the long-term decoding stability when only a few days of training data were available.

## Data Availability Statement

The datasets generated for this study are available on request to the corresponding author.

## Ethics Statement

The animal study was reviewed and approved by the Institutional Animal Care and Use Committee of the Taipei Medical University.

## Author Contributions

S-HY, C-HK, and Y-YC designed the project, organized the entire research. Y-CL, H-YL, and S-HL conceived the experiments. CC, J-WH, H-LW, C-FW, and C-CK conducted the experiments. CC, S-HY, Y-CL, H-LW, K-YC, and C-FW implemented code from software designed and planned. CC, S-HL, J-WH, H-YL, and Y-HL analyzed the results. S-HY, CC, and Y-YC wrote the manuscript. All authors discussed the results and reviewed on the manuscript.

### Conflict of Interest

The authors declare that the research was conducted in the absence of any commercial or financial relationships that could be construed as a potential conflict of interest.

## Supplementary Material

Supplemental Video The supplemental video showed forelimb reaching for lever pressing and simultaneous neural recording in M1.

## Abbreviations

MI: brain machine interface
ECPNN-EF: evolutionary constructive and pruning neural network with error feedback
NN: neural network
RNN: recurrent neural network
ESN: echo-state network
TDNN: time-delay neural network
EA: evolutionary algorithm
ECPA: evolutionary constructive and pruning algorithm
CBP: cluster-based pruning
ABSS: age-based survival selection
BPTT: backpropagation through time
ECPNN-EFWC: ECPNN-EF only with CBP
ECPNN-EFWA: ECPNN-EF only with ABSS
RNN-EF: fully-connected RNN with error feedback
ECPNN: ECPNN-EF without error-correction learning
PCNN: partially connected NN
FCNN: fully connected neural network.
