# Supplementary material for: Inhibition of Long-Term Variability in Decoding Forelimb Trajectory Using Evolutionary Neural Networks With Error-Correction Learning
Source: Front Comput Neurosci. 2020 Mar 31;14:22. doi: 10.3389/fncom.2020.00022 (PMC7136463; doi:10.3389/fncom.2020.00022)
Supplement: Supplementary file 2 [file Data_Sheet_1.docx]

Supplementary Material

**Inhibition of Long-Term Variability in Decoding Forelimb Trajectory using Evolutionary Neural Networks with Error-Correction Learning**

Shih-Hung Yang^1^, Han-Lin Wang^2^, Yu-Chun Lo^3^, Hsin-Yi Lai^4, 5^, Kuan-Yu Chen^2^, Yu-Hao Lan^2^, Ching-Chia Kao^6^, Chin Chou^7^, Sheng-Huang Lin^8, 9^, Jyun-We Huang^1^, Ching-Fu Wang^2^, Chao-Hung Kuo^2, 10, 11^, and You-Yin Chen ^2, 3, *^

^1^ Department of Mechanical Engineering, National Cheng Kung University, No. 1 University Rd., Tainan, Taiwan 70101, R.O.C.

^2^ Department of Biomedical Engineering, National Yang Ming University, No.155, Sec.2, Linong St., Taipei, Taiwan 11221, R.O.C.

^3^ The Ph.D. Program for Neural Regenerative Medicine, College of Medical Science and Technology, Taipei Medical University, No. 250 Wu-Xing St., Taipei 11031, Taiwan, R.O.C.

^4^ Department of Neurology of the Second Affiliated Hospital, Interdisciplinary Institute of Neuroscience and Technology, Key Laboratory of Medical Neurobiology of Zhejiang Province, Zhejiang University School of Medicine, No.268, Kaixuan Rd., Hangzhou 310029, China

^5^ Key Laboratory of Biomedical Engineering of Ministry of Education, Qiushi Academy for Advanced Studies, College of Biomedical Engineering and Instrument Science, Zhejiang University, No. 38 Zheda Rd., Hangzhou 310027, China

^6^ Research Center for Information Technology Innovation, Academia Sinica, No. 128, Sec. 2, Academia Rd., Taipei 11529, Taiwan, R.O.C.

^7^ Department of Regulatory & Quality Sciences, University of Southern California, No. 1540 Alcazar St., CHP 140, Los Angeles, CA 90033, U.S.A.

^8^ Buddhist Tzu Chi Medical Foundation, Department of Neurology, Hualien Tzu Chi Hospital, Hualien, No. 707, Sec. 3, Chung Yang Rd., Hualien 97002, Taiwan, R.O.C.

^9^ Department of Neurology, School of Medicine, Tzu Chi University, No. 701, Sec. 3, Zhongyang Rd., Hualien 97004, Taiwan, R.O.C.

^10^ Department of Neurosurgery, Neurological Institute, Taipei Veterans General Hospital, No. 201, Sec. 2, Shipai Rd., Taipei, Taiwan11217, R.O.C.

^11^ Department of Neurological Surgery, University of Washington, No.1959 NE Pacific St., Seattle, WA 98195-6470, U.S.A.

^*^Correspondence should be addressed to the following:

You-Yin Chen, Department of Biomedical Engineering, National Yang Ming University, No.155, Sec.2, Linong St., Taipei, Taiwan 11221, R.O.C.

E-mail: irradiance@so-net.net.tw

#### *Note 1. Backpropagation Through Time*

The backpropagation through time (BPTT) algorithm first decomposed the weights in **Eq. (S1)** into three weight matrices ($\boldsymbol{W}_{hx}, \boldsymbol{W}_{hh},$ and $\boldsymbol{W}_{oh}$) where *h*, *x*, and *o* represented hidden state, input, and output neurons, respectively. Note that $\boldsymbol{W}_{hh}$ represented recurrent weight matrix. The hidden neuron model was defined as follows:

$\boldsymbol{h}_{t}=\boldsymbol{W}_{hx}\boldsymbol{x}_{t}+\boldsymbol{W}_{hh}\boldsymbol{h}_{t-1}$. (S1)

The gradient of the loss function in **Eq. (S2)** with respect to $\boldsymbol{W}_{hx}$ and $\boldsymbol{W}_{hh}$ were computed as:

$\frac{\partial L}{\partial\boldsymbol{W}_{hx}}=\sum_{t=1}^{T} \mathrm{prod}\left( \frac{\partial L}{\partial\boldsymbol{O}_{t}},\boldsymbol{W}_{oh},\frac{\partial\boldsymbol{h}_{t}}{\partial\boldsymbol{W}_{hx}} \right)$, (S2)

$\frac{\partial L}{\partial\boldsymbol{W}_{hh}}=\sum_{t=1}^{T} \mathrm{prod}\left( \frac{\partial L}{\partial\boldsymbol{O}_{t}},\boldsymbol{W}_{oh},\frac{\partial\boldsymbol{h}_{t}}{\partial\boldsymbol{W}_{hh}} \right)$, (S3)

where $\mathrm{prod}\left( \cdot\right)$ represented the product of two or more matrices and:

$\frac{\partial\boldsymbol{h}_{t}}{\partial\boldsymbol{W}_{hx}}=\sum_{j=1}^{t} \left( \boldsymbol{W}_{hh}^{T} \right)^{t-j}\boldsymbol{x}_{j}$, (S4)

$\frac{\partial\boldsymbol{h}_{t}}{\partial\boldsymbol{W}_{hh}}=\sum_{j=1}^{t} \left( \boldsymbol{W}_{hh}^{T} \right)^{t-j}\boldsymbol{h}_{j}$. (S5)

The weights were then updated using **Eq. (4)**.

#### *Note 2. Evolutionary Constructive and Pruning Neural Network with Error Feedback (ECPNN-EF) Training Algorithm*

The proposed decoder, ECPNN-EF, was evolved according to the training algorithm (major steps summarized in **Figure S1**; details provided below).


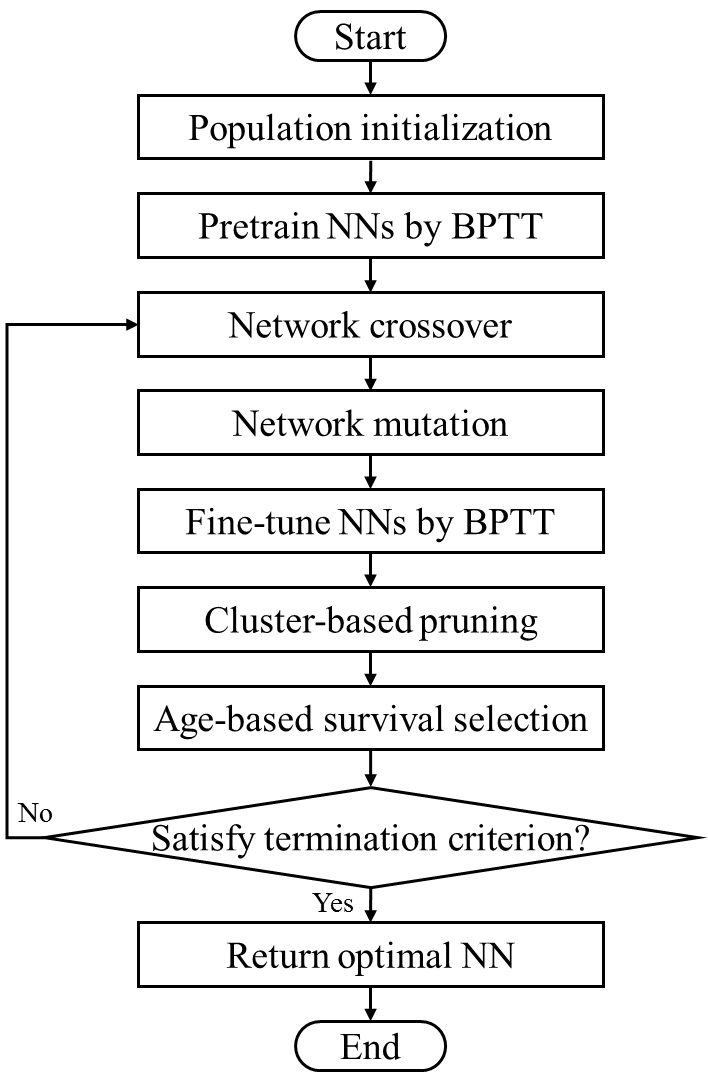


**Figure S1.** ECPNN-EF training algorithm flow chart.

*Initialization phase*

1. Generate initial $N_{p}$ parent neural networks (NNs) with a partially connected topology.
2. Pretrain the NN weights using the BPTT algorithm [[1](#_ENREF_1)] for $\varphi$ epochs using the training set, then calculate their fitness (i.e., the inverse of the loss calculated by **Eq. (2)**).

*Reproduction phase*

1. Use tournament selection to select two parent NNs. Perform network crossover to merge the two parent NN substructures to produce an offspring NN with a crossover probability, $p_{c}$.
2. Perform network mutation of the offspring NN to generate a new connection with a mutation probability, $p_{m}$.
3. Return to ***Step 3*** and execute the subsequent steps until $N_{p}$ offspring NNs have been reproduced.
4. Fine-tune the offspring NN weights using BPTT for *φ* epochs using the training set, then determine their fitness.
5. Conduct cluster-based pruning (CBP) on the offspring NNs.
6. Select $N_{p}$ NNs as the parent NNs of the next generation by applying age-based survival selection (ABSS) to the parent NNs and their offspring NNs.
7. Return to ***S******tep 3*** and execute the subsequent steps until the termination criterion is satisfied using the validation set or until the maximum number of generations, *G*, is reached.
8. Select the optimal NN with partially connected topology as the neural decoder.

The parameters used in the ECPNN-EF training algorithm are listed in **Table S1**. Note that $p_{c}$ and $p_{m}$ were determined by analyzing the effects of crossover and mutation probabilities on decoding performance, respectively.

**Table S1.** ECPNN-EF training algorithm parameters, where $m$ is the dimension of the input vector.

| $N_{p}$ | *φ* | *G* |
| --- | --- | --- |
| $2m$ | 500 | 100 |

#### *Note 3. Population Initialization*

The initial population consisted of a set of NNs that possessed one hidden layer and were produced following these steps:

1. Generate one neuron in a hidden layer.
2. Randomly generate a single connection from one error-related input neuron to one hidden neuron or to the output neuron.
3. Randomly generate a single connection from one non-error-related input neuron to a hidden neuron.
4. Randomly generate a skip connection from one non-error-related input neuron to the output neuron.
5. Generate a single recurrent connection of one hidden neuron with a probability of 0.5. All weights were initialized in the [−1.0, 1.0] range.

As shown in **Figure 5**, the error-related input neuron processed the previous prediction error, whereas the non-error-related input neuron processed the instantaneous firing rate of each unit. Generating a connection with the error-related input neuron ensured that all initial NNs possessed error feedback at the beginning of the NN evolution.

#### *Note 4. Network Crossover*

Network crossover is a constructive operator that increases the number of hidden neurons in NNs to explore a wider structural search space and increases processing capabilities. It was performed twice independently using a tournament selection to obtain two parent NNs. The tournament selection first randomly selected two parent NNs from the population, then reserved the NN with the best fitness as one parent NN. The two parent NN substructures were merged to reproduce an offspring NN. **Figure S2** illustrates an example of network crossover, where *NN_a_* and *NN_b_* were parent NNs and *NN_c_* was their offspring NN. The input–output relationship of *NN_a_* was given by:

$y^{a}=w_{1}^{a}\cdot u_{1}+w_{5}^{a}\cdot h^{a}$, (S6)

where $y$, $u$, $h$, and $w$ are the output neuron, input neuron, hidden neuron, and weight, respectively. Note that the hidden neuron consisted of a recurrent connection and $u_{5}$ represented the error feedback. Only the weights that were connected to the output neuron are displayed because the remaining weights were unchanged after network crossover. The superscript denotes its network index. The ground truth velocity was scaled to the [−1.0, 1.0] range. The input–output relationship of *NN_b_* was given by:

$y^{b}=w_{6}^{b}\cdot h_{1}^{b}+w_{7}^{b}\cdot h_{2}^{b}+w_{3}^{b}\cdot u_{4}$. (S7)

Note that $w_{3}^{b}$ is a skip connection. The network crossover merged the *NN_a_* and *NN_b_* substructures to produce an offspring, *NN_c_*. Therefore, the input–output relationship of *NN_c_* was given by:

$y^{c}=\frac{1}{2}w_{1}^{a}\cdot u_{1}+\frac{1}{2}w_{5}^{a}\cdot h^{a}+\frac{1}{2}w_{6}^{b}\cdot h_{1}^{b}+\frac{1}{2}w_{7}^{b}\cdot h_{2}^{b}+\frac{1}{2}w_{3}^{b}\cdot u_{4}.$ (S3)

Notably, half of the weights that were connected to the offspring NN output neurons were from the parent NNs. The offspring NN retained other weights from the parent NNs. The crossover probability, $p_{c}$, was specified to determine the chance of performing network crossover. If $p_{c}$ was smaller than a random number, then the two parent NNs were simply copied as two offspring NNs.

**
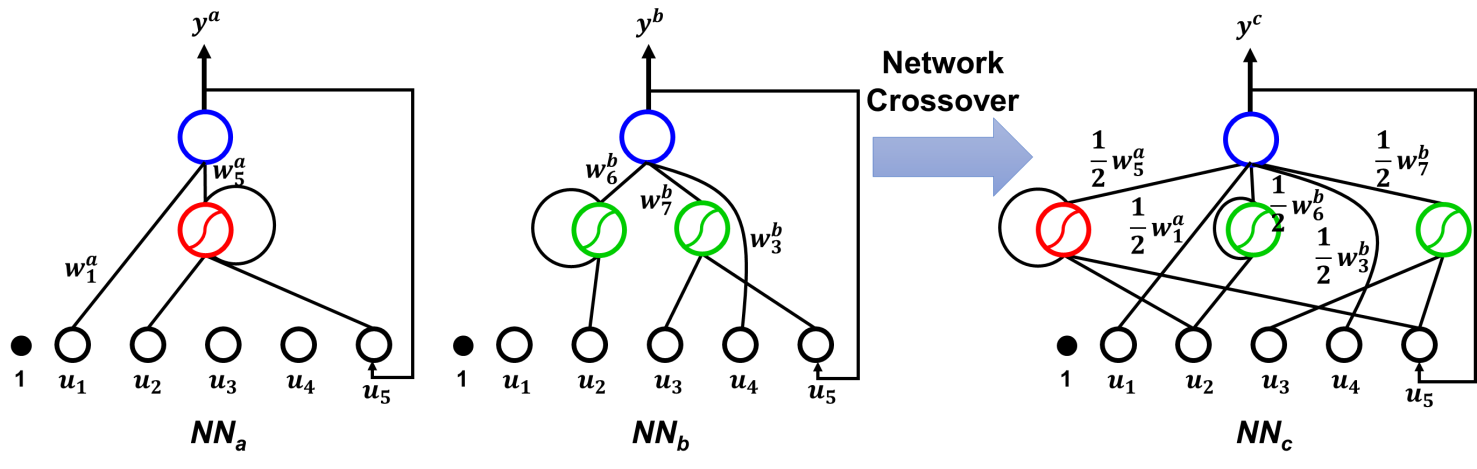
**

**Figure S2.** Example of network crossover. Network crossover merged the substructures of the parents, *NN_a_* and *NN_b_*, to reproduce an offspring, *NN_c_*.

#### *Note 5. Network Mutation*

Network mutation is a constructive operator that was used to generate connections. Network mutation allowed hidden and output neurons to process more information from input neurons, and thus improved the NN’s prediction capability. A new connection with a [−0.01, 0.01] weight range was randomly generated in the network. **Figure S3** illustrates the network mutation, where a new connection, $w_{8}^{b}$, was added between the first input neuron and the first hidden neuron. A mutation probability, $p_{m}$, was specified to determine the chance of performing network mutation. If $p_{m}$ was smaller than a random number, then the NN was not mutated. After network *crossover* and *mutation*, the network was fine-tuned using BPTT to improve its decoding performance.


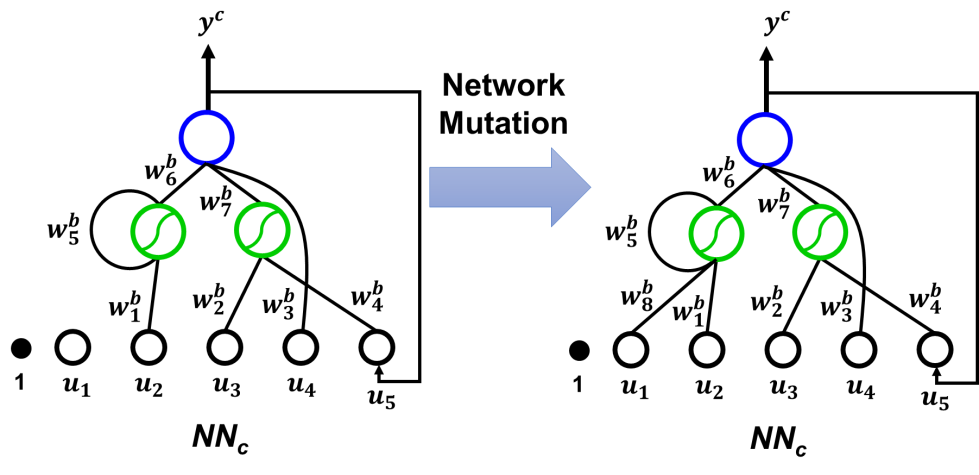


**Figure S3.** Example of network mutation. Network mutation randomly generated one connection in the network.

#### *Note 6. Cluster-based Pruning (CBP)*

CBP is a pruning operator that was used to retain significant hidden neurons and eliminate insignificant ones. This helped to minimize creation of a network with an excessively large and complex structure. The main purpose of the CBP was to separate hidden neurons into two groups, better and worse, then randomly delete the hidden neurons in the worse group. CBP was carried out in three steps. The first step calculated the significance of the *i*^th^ hidden neuron as:

$\sigma_{i}=\sqrt{\left| w_{i} \right|}$, (S8)

where $w_{i}$ is the output weight of the *i*^th^ hidden neuron. The second step categorized the hidden neurons into two groups: better and worse. The hidden neuron with the greatest significance was assigned the prototype role for the better group, whereas the hidden neuron with the least significance was assigned the prototype role for the worse group. The remaining hidden neurons were categorized into the better group according to how closely their significance aligned to the better prototype. Hidden neurons that were not close to the better prototype were categorized into the worse group. The third step retained the better neurons and deleted the worse neurons in a stochastic manner. For each neuron in the worse group, the neuron was deleted if a random number was larger than 0.5 but was retained otherwise.

#### *Note 7. Age-based Survival Selection (ABSS)*

ABSS selected $N_{p}$ NNs as the parent NNs for the next generation based on the age of the NN. Younger NNs tended to have a partially connected topology, whereas older NNs tended to have a fully connected topology because of the use of network mutation. ABSS potentially reserved younger NNs to achieve a better processing capability per connection. ABSS was carried out in two steps. The first step used traditional tournament selection to choose $N_{p}$ NNs from the parent and offspring NNs. The NN age was defined as the number of generations that it survived in the population. The health index was defined as follows:

$H_{j}=\left( 1-\frac{1}{{Age}_{j}} \right)^{2}$, (S9)

where ${Age}_{j}$ is the age of the *j*^th^ NN. The second step performed survival selection. If $H_{j}$ was larger than a uniform random number, then the *j*^th^ NN was removed and replaced by an initial NN generated by the population initialization in *Note 3*. Otherwise, the *j*^th^ NN was retained for the next generation.

#### *Note 8. Criteria for Selecting Neurons into the Neural Decoder*

Our empirical data indicated that neural activity synchronization was associated with specific movement states, such as lever pressing in the water-reward task. Therefore, the causal relationship between recorded M1 neural signals can provide more discriminative information for decoding the laterality of forelimb movement [[2](#_ENREF_2), [3](#_ENREF_3)]. Based on the causal analysis from the peristimulus time histogram, we found a strong relationship in neuron-motor response. The maximum firing rate from the ensemble neurons was consistently present at the third time-bin (with 99-ms lag) prior to the lever-pressing event. In this study, the recorded neurons were generally capable of firing more than once at the third time-bin for all behavioral trials per day. This bin was considered to be associated with forelimb movement and was thereby enrolled as the input of neural features to the neural decoder. **Fig. S4** shows the average neuronal firing rate of each sorted unit at the third time-bin during the 12 experimental days. During the first 2 days, all sorted neuron firing rates met the experience criteria (spike count > 1 at the third time-bin) due to less variation in the recording condition. However, these neuronal spiking signals suffered considerable information loss across the subsequent 10 days, including degradation of spike firing or unit loss. This may have been due to inflammatory tissue reaction to neural implants [[4](#_ENREF_4)] or micromotion around the electrodes [[5](#_ENREF_5)] leading to recording failure. The number of sorted units was inconsistent from day-to-day. Thus, the input number of neural features to the neural decoder was determined by the minimum number of available units across the experimental days. Under circumstances where the sorted neurons outnumbered the minimum, an equivalent number of available neurons were selected in descending order of spike count at the third time-bin. For example, the minimum number of available units recorded from Rat #11 was 8 on the 10^th^ day. Therefore, eight neuronal features were used as the input to the decoder. If more than eight available units were present from Rat #11 on any given day, those units with the top eight ranked firing rates were selected for the neural decoder. These units were not necessarily consistent from day-to-day.


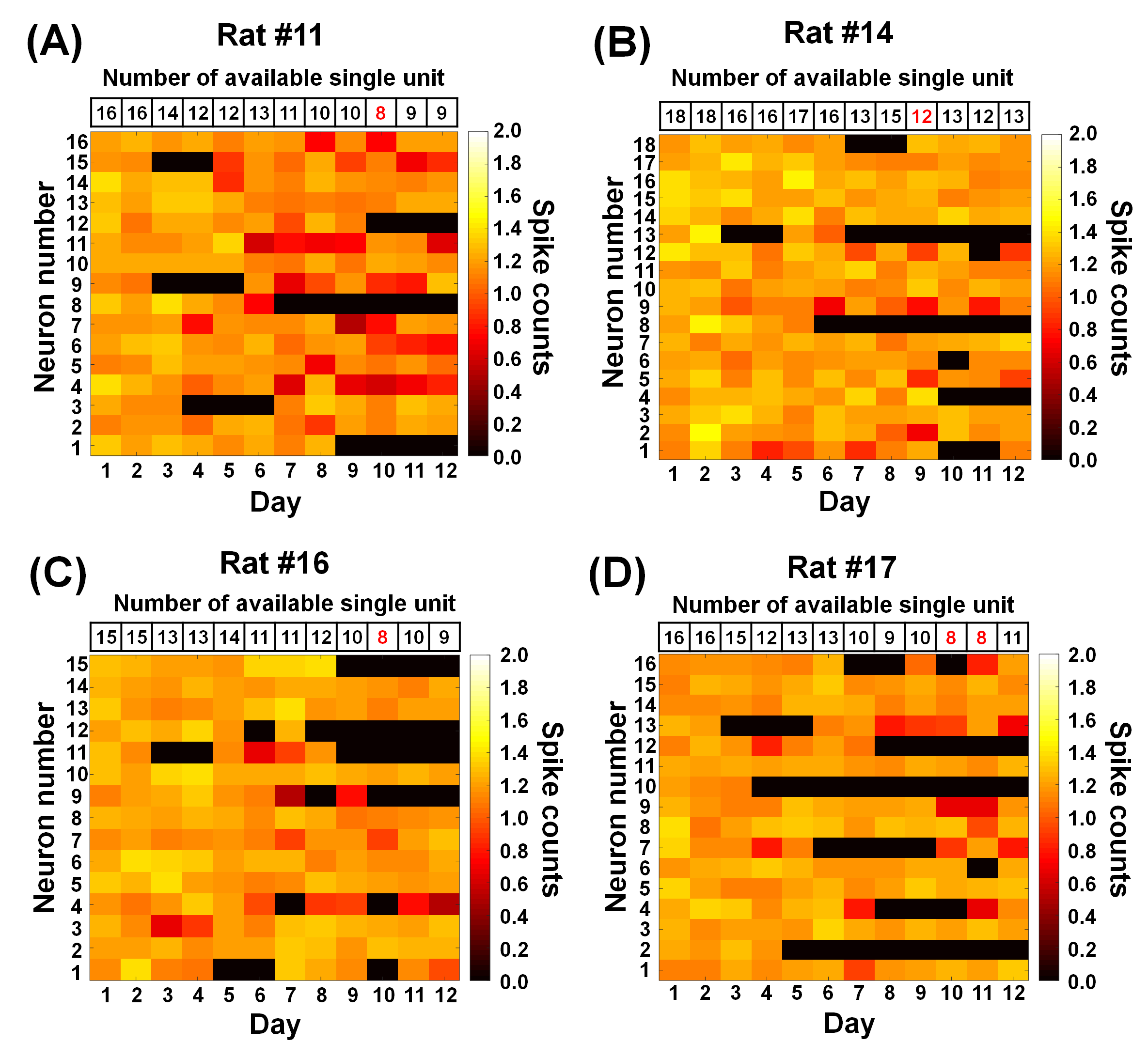


**Fig. S4. Average neuronal spike firing at third time-bin in each sorted neuron among the experimental days.** The vertical axis represents the sorted neurons with mean firing rates greater than one at the third time-bin. The horizontal axis represents the experimental days—the first 2 days and the subsequent 10 days were used for training and testing, respectively. Top: the number of the available single units used as the input to neural decoder per day

#### *Note 9. Analysis of Simple Main Effects of Crossover and Mutation Probabilities*

#### We validated the simple effect of *p_m_* across seven levels with the *p_c_* level fixed at 0.75 and 0.8. *Post-hoc* analysis was performed based on the estimated marginal means of the correlation coefficient (*r*). Bonferroni correction was used to adjust the *P*-value for multiple comparisons (Table S2). The greatest *r* value was observed when *p_m_* = 0.75 and *p_c_* = 0.75 (*r* = 0.912), which was significantly greater than the *r* value when *p_m_* = 0.75 and *p_c_* = 0.8. Therefore, the near-optimum solution settings for the algorithm were *p_c_* = 0.75 and *p_m_* = 0.75.

**Table S2**. The simple effect of *p_m_* across seven levels with the *p_c_* level fixed at 0.75 and 0.8.

| *p_m_* | Correlation coefficient (*r*) | | Mean difference of *r* (*p_c_*_0.75_-*p_c_*_0.8_) | *F* | *Adjusted P* |
| --- | --- | --- | --- | --- | --- |
|  | ***p_c_* = 0.75** | ***p_c_* = 0.8** |  |  |  |
| 0.6 | 0.812 ± 0.039 | 0.803 ± 0.009 | 0.008 | 0.271 | 0.607 |
| 0.65 | 0.833 ± 0.017 | 0.840 ± 0.025 | −0.007 | 0.209 | 0.651 |
| 0.7 | 0.823 ± 0.026 | 0.867 ± 0.023 | −0.044 | 7.606 | 0.01^*^ |
| 0.75 | 0.912 ± 0.019 | 0.824 ± 0.016 | 0.087 | 29.900 | 0.0001^*^ |
| 0.8 | 0.842 ± 0.014 | 0.788 ± 0.017 | 0.054 | 11.320 | 0.002^*^ |
| 0.85 | 0.841 ± 0.021 | 0.823 ± 0.018 | 0.018 | 0.289 | 0.582 |
| 0.9 | 0.806 ± 0.027 | 0.810 ± 0.025 | −0.004 | 0.317 | 0.544 |

*Significant difference after Bonferroni correction for multiple testing (*P* < 0.05).

#### *Note 10. Comparison of ECPNN-EF, RNN-EF, and ECPNN Decoding Performance*

We applied a mixed model ANOVA with three decoders (ECPNN-EF, RNN-EF, and ECPNN) as fixed factors and time as a repeated factor. The *P*-value was adjusted using the Bonferroni multiple comparison correction. The *post-hoc* analysis showed that the ECPNN-EF decoder had a significantly higher correlation coefficient (***r***) than the RNN-EF and ECPNN decoders over the 10 test days (**Table S3**).

**Table S3**. The *post-hoc* analysis comparing decoding performances of the three decoders over the 10 test days.

| *Test day* | *r* (Mean ± SD) | | | | *Adjusted P* |
| --- | --- | --- | --- | --- | --- |
| 1 | ***ECPNN-EF*** | 0.912 ± 0.018 | ***ECPNN*** | 0.878 ± 0.018 | 0.021^*^ |
|  |  |  | ***RNN-EF*** | 0.793 ± 0.029 | 0.034^#^ |
| 2 | ***ECPNN-EF*** | 0.860 ± 0.027 | ***ECPNN*** | 0.771 ± 0.019 | 0.040^*^ |
|  |  |  | ***RNN-EF*** | 0.738 ± 0.035 | 0.023^#^ |
| 3 | ***ECPNN-EF*** | 0.856 ± 0.036 | ***ECPNN*** | 0.816 ± 0.018 | 0.028^*^ |
|  |  |  | ***RNN-EF*** | 0.719 ± 0.061 | 0.018^#^ |
| 4 | ***ECPNN-EF*** | 0.878 ± 0.019 | ***ECPNN*** | 0.749 ± 0.035 | 0.034^*^ |
|  |  |  | ***RNN-EF*** | 0.763 ± 0.044 | 0.029^#^ |
| 5 | ***ECPNN-EF*** | 0.842 ± 0.021 | ***ECPNN*** | 0.764 ± 0.041 | 0.018^*^ |
|  |  |  | ***RNN-EF*** | 0.742 ± 0.078 | 0.024^#^ |
| 6 | ***ECPNN-EF*** | 0.831 ± 0.023 | ***ECPNN*** | 0.684 ± 0.052 | 0.039^*^ |
|  |  |  | ***RNN-EF*** | 0.672 ± 0.063 | 0.041^#^ |
| 7 | ***ECPNN-EF*** | 0.807 ± 0.025 | ***ECPNN*** | 0.550 ± 0.074 | 0.044^*^ |
|  |  |  | ***RNN-EF*** | 0.613 ± 0.048 | 0.048^#^ |
| 8 | ***ECPNN-EF*** | 0.773 ± 0.005 | ***ECPNN*** | 0.577 ± 0. 084 | 0.045^*^ |
|  |  |  | ***RNN-EF*** | 0.639 ± 0.052 | 0.042^#^ |
| 9 | ***ECPNN-EF*** | 0.779 ± 0.019 | ***ECPNN*** | 0.467 ± 0.140 | 0.039^*^ |
|  |  |  | ***RNN-EF*** | 0.598 ± 0.022 | 0.035^#^ |
| 10 | ***ECPNN-EF*** | 0.740 ± 0.042 | ***ECPNN*** | 0.413 ± 0.158 | 0.036^*^ |
|  |  |  | ***RNN-EF*** | 0.549 ± 0.040 | 0.049^#^ |

^*^  and ^#^ indicated significant differences in ***r*** relative to the decoder of ECPNN-EF, analyzed by the mixed model ANOVA with after Bonferroni correction for multiple testing (*P* < 0.05).

#### *Note 11. Comparison between the ECPNN-EF and SIR-EF decoders*

An SIR-EF was implemented based on an SIR linear neural decoder [[3](#_ENREF_3)] to fairly compare to the ECPNN-EF algorithm. Data from the present study generated average predicted trajectories and decoding performances for ECPNN-EF and SIR-EF (**Fig. S5** and **Fig. S6**, respectively). SIR-EF’s decoding performance was ***r*** = 0.805 ± 0.021 on Test Day 1 and 0.753 ± 0.035 on Test Day 2. Notably, its decoding performance rapidly decreased day-over-day to ***r*** = 0.352 ± 0.095 on Test Day 10. Conversely, the ECPNN-EF decoder achieved a better and more stable decoding performance across the 10 days compared to linear decoding using SIR-EF.


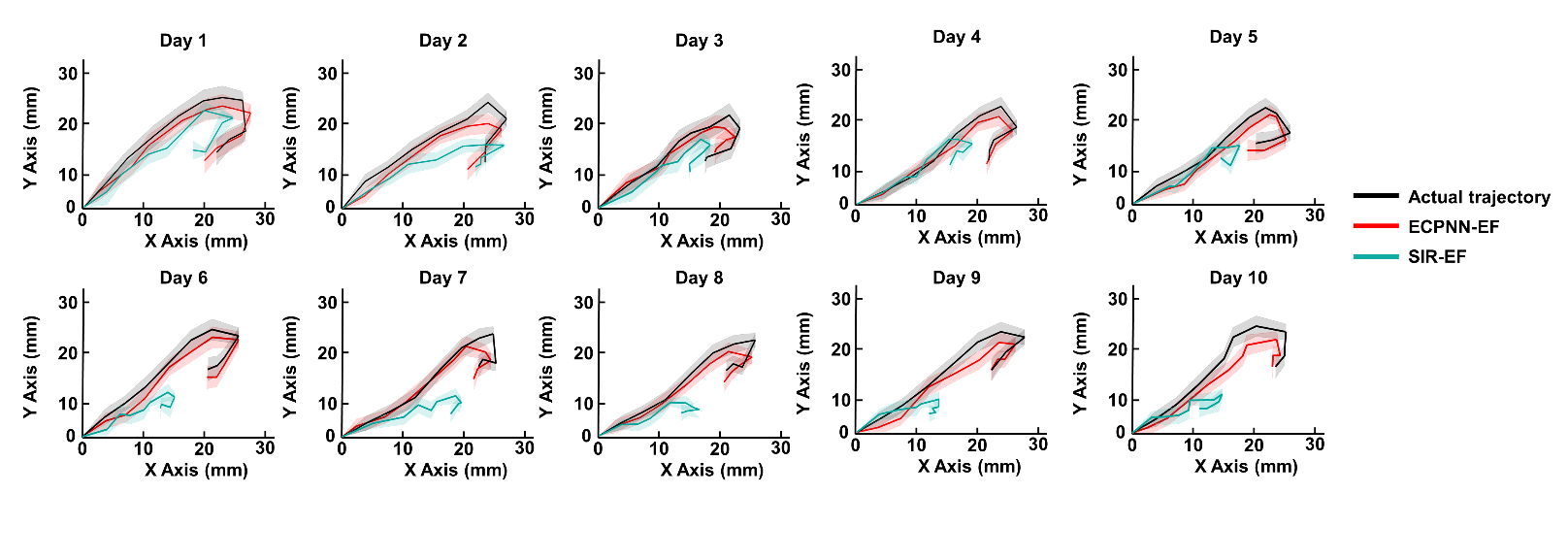


**Fig. S5**. **Reconstructed trajectories predicted by the ECPNN-EF and SIR-EF decoders across 10 testing days in Rat #16**. The shaded region represents the standard deviation of the average predicted trajectories of the ECPNN-EF (red) and SIR-EF (cyan) decoders. The black curve represents the actual trajectory.


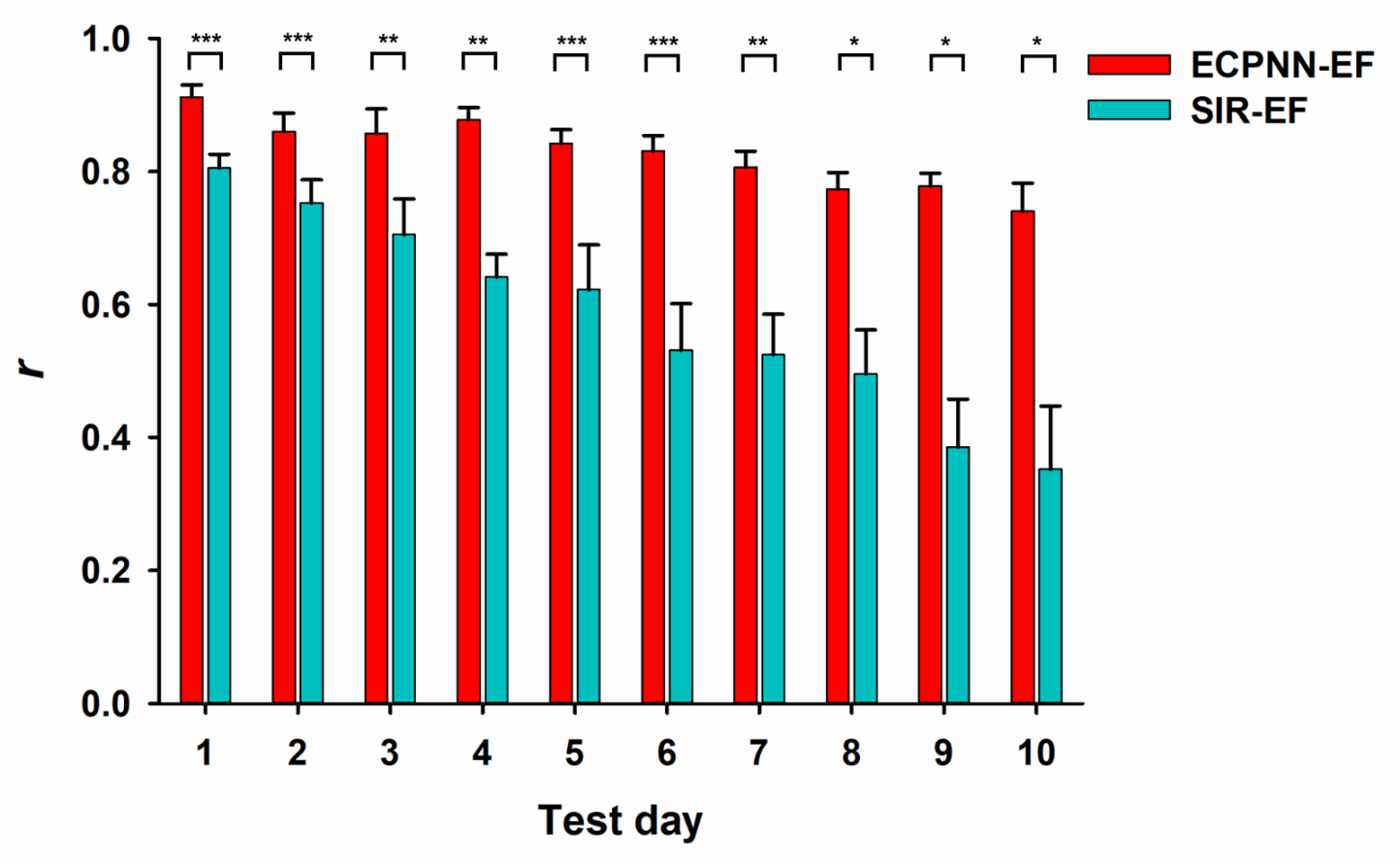


**Fig. S6**. **Comparing ECPNN-EF and SIR-EF decoding performances.** Comparing the daily ***r*** values of the ECPNN-EF and SIR-EF decoders. The decoding performance of ECPNN-EF was significantly higher than that of the SIR-EF on all test days. Further, the corresponding ***r*** variation was smaller for the ECPNN-EF than for SIR-EF across days. Significant levels generated by a Mann–Whitney U Test at **P* < 0.05, ***P* < 0.01, and ****P* < 0.001.

References:

1. Werbos, P.J., *Generalization of backpropagation with application to a recurrent gas market model.* Neural Networks, 1988. **1**(4): p. 339-356.

2. Wu, W., et al., *Modeling and decoding motor cortical activity using a switching Kalman filter.* IEEE Transactions on Biomedical Engineering, 2004. **51**(6): p. 933-942.

3. Yang, S.-H., et al., *A Sliced Inverse Regression (SIR) decoding the forelimb movement from neuronal spikes in the rat motor cortex.* Frontiers in Neuroscience, 2016. **10**: p. 556.

4. Barrese, J.C., et al., *Failure mode analysis of silicon-based intracortical microelectrode arrays in non-human primates.* Journal of Neural engineering, 2013. **10**(6): p. 066014-066014.

5. Sussillo, D., et al., *Making brain-machine interfaces robust to future neural variability.* Nature Communications, 2016. **7**: p. 13749-13749.
